# Supplementary material for: Greatwall promotes cell transformation by hyperactivating AKT in human malignancies
Source: eLife. 2015 Nov 27;4:e10115. doi: 10.7554/eLife.10115 (PMC4733044; doi:10.7554/eLife.10115)
Supplement: Supplementary file 1. — DOI: http://dx.doi.org/10.7554/eLife.10115.026 [file elife-10115-supp1.doc]

**Supplementary File 1:** Antibodies used in the study

| **TARGET** | **SPECIES** | **SUPPLIER** |
| --- | --- | --- |
| pAKT T308 | Rabbit | Cell Signalling (cat 9275) |
| pGSK3 α/β S21/S9 | Mouse | Cell Signalling (cat 8566) |
| Human GWL | Rabbit | Burgess et al. PNAS (2010) |
| β-Tubulin | Mouse | Hybridoma E7 |
| RAS | Mouse | Santa Cruz (cat SC-63) |
| HA | Mouse | Roche 12CA5 (cat 11583816001) |
| Phospho-P44/42 MAPK | Mouse | Cell Signalling (cat 9106) |
| Human ENSA | Rabbit | This study, full-length recombinant antigen, affinity purified |
| PHLPP1 | Rabbit | Bethyl (cat A300-660A) |
| RICTOR | Rabbit | Cell Signaling (cat 2114) |
| B56γ | Mouse | Santa Cruz (cat 374380) |
| pAKT S473 | Rabbit | Cell Signaling (cat 4060) |
| Human ARPP19 | Rabbit | Gharbi-Ayachi et al. Science (2010) |
| β-Catenin | Rabbit | Cell Signalling (cat 9562) |
| Red Fluorescent Protein | Rat | ChromoTek (cat 5F8) |
