# Supplementary material for: Greatwall promotes cell transformation by hyperactivating AKT in human malignancies
Source: eLife. 2015 Nov 27;4:e10115. doi: 10.7554/eLife.10115 (PMC4733044; doi:10.7554/eLife.10115)
Supplement: Supplementary file 2. — DOI: http://dx.doi.org/10.7554/eLife.10115.027 [file elife-10115-supp2.docx]

**Supplementary File 2:** Plasmids used in the study

| **Construct** | **Restriction site** | **SUPPLIER** |
| --- | --- | --- |
| pCS2-HA-hGWL | EcoRV | Vigneron et al. (MCB 2011) |
| pCS2-HA-hGWL-K72M | EcoRV | Vigneron et al. (MCB 2011) |
| pCS2-HA-hGWL-G44S | EcoRV | Vigneron et al. (MCB 2011) |
| pCS2-xB56γ | EcoRV | Imagenes, PCR from a pCMV Sport6 plasmid |
| pMXs-hGWL | BamHI/XhoI | This study, cloned from the pCS2-HA-hGWL-K72M |
| pMXs-hGWL-K72M | BamHI/XhoI | This study, cloned from the pCS2-HA-hGWL-K72M |
| pMXs-hGWL-G44S | BamHI/XhoI | This study, obtained from the pCS2-HA-hGWL-G44S |
| pMXs-hB55δ-RFP |  | The CCSB Human ORFEOME Collection |
| pBABE-V12 Ras | BamHI/HindIII | Cloned from plasmid pECEG-V12-Ras (Roux et al., 1997) |
| pSIREN-shGWL | BamHI/EcoRI | Sequence 1 from Burgess et al PNAS (2010) |
| pSR-GFP-shRICTOR | HindIII/BglII | Generous gift from A. Sirvant. |
| pMXs-HA-hARPP19 | BamHI/XhoI | This study, obtained from a pCS2-HA-hArpp19 in which cDNA of hArpp19 was cloned in blunt by EcoRV digestion |
| pMXs-HA-hARPP19 S62A |  | This study. Obtained by site-directed mutagenesis from the pMXs-HA-hArpp19 plasmid |
| pMXs-hENSA | BamHI/XhoI | This study, from a pGEX-ENSA plasmid from Gharbi-Ayachi et al. Science (2010) |
| pMXs-hENSA S67A |  | This study. Obtained by site-directed mutagenesis |
| pZIP-SVX-SV40T |  |  |
| pBABE-hTERT | EcoRI | Gire et al. (Oncogene 2000) |
| pcDNA-HA-PHLPP1 |  | Addgene (ID22404) |
